# COVID-19-related cardiovascular disease risk due to weight gain: a nationwide cohort study

**DOI:** 10.1186/s40001-023-01569-7

**Published:** 2024-01-02

**Authors:** Su Kyoung Lee, Yohwan Lim, Seogsong Jeong, Hyun Wook Han

**Affiliations:** 1 Institute of Health and Environment, Seoul National University, Seoul, Korea; 2 Department of Biomedical Informatics, CHA University School of Medicine, CHA University, 335 Pangyo-Ro, Seongnam, 13448 Republic of Korea

**Keywords:** Body-mass index, BMI, Cardiovascular disease, Obesity, Weight control

## Abstract

**Background:**

Cardiovascular disease (CVD) is a significant contributor to morbidity and mortality worldwide, with CVD and post-acute COVID-19 associated CVD increasing. It remains unknown whether COVID-19 patients with weight gain are at a high risk for CVD events. Therefore, the primary objective of this study is to investigate the association between weight control and the risk of CVD following COVID-19.

**Methods:**

The study included 2,024,728 adults who participated in two rounds of health screening between 2017 and 2020. The final cohort, which included 70,996 participants in the COVID-19 group and 212,869 participants in the control group. The adjusted hazard ratio of BMI change to CVD risk was calculated using Cox proportional hazards regression.

**Results:**

We identified a total of 2869 cases of CVD (861 events for COVID-19 group and 2,008 events for the control group). Compared to individuals with a stable BMI, COVID-19 patients without obesity had an increased risk of CVD (adjusted hazard ratio [aHR] = 2.28; 95% confidence interval [CI], 1.15–4.53; p-value = 0.018). Additionally, non-COVID-19 patients with obesity also exhibited a higher risk of CVD (aHR = 1.58; 95% CI, 1.01–2.47; p-value = 0.046).

**Conclusion:**

In conclusion, people who gained weight during the pandemic, regardless of their weight category, had a significantly higher risk of CVD associated with COVID-19 compared to those who maintained their weight before the pandemic.

**Supplementary Information:**

The online version contains supplementary material available at 10.1186/s40001-023-01569-7.

## Introduction

Cardiovascular disease (CVD) is one of the largest contributors to morbidity and mortality worldwide [1]. CVD accounts for 40.8 million disability-adjusted life years (DALYs) each year in the Americas, which measure the years of healthy life lost due to disease or death [2], and CVD, including coronary heart disease (CHD) and stroke associated with post-acute COVID-19, is steadily increasing [3]. A higher risk of death and incident CVD from 30 days after the diagnosis of COVID-19 until 4 months was found associated with COVID-19 [4, 5].

Along with traditional and novel risk factors for COVID-19-related CVD, obesity is a major public health concern associated with an increased risk of CVD [6]. It has been a growing epidemic over the past 50 years, resulting in enormous healthcare costs of over $700 billion annually and a high association with developing diabetes, CVD, hypertension, and hyperlipidemia [7]. The updated American Heart Association (AHA) Scientific Statement on Obesity and CVD and the European Heart Association (ESC) Expert Position Report highlight the detrimental effects of obesity on cardiovascular health, independently of other cardiovascular risk factors [6]. Although the underlying biological mechanisms and predisposing risk factors that lead to cardiovascular sequelae in COVID-19 survivors are not yet fully understood [8], a prospective cohort study in the UK identified systemic inflammation and obesity as possible factors associated with long-term COVID-19 sequelae [9].

The obesity paradox is supported by empirical studies demonstrating that among patients with acute myocardial infarction, atrial fibrillation, and coronary artery disease, people with overweight or obesity have a lower risk of death from CVD than those with normal weight [10–12]. These studies suggest that obesity may have a protective effect on some cardiovascular outcomes, but the mechanisms and implications of this effect are not fully understood. There are multiple types of weight gain, such as an increase in body fat distribution and weight gain after smoking cessation rather than just an increase in BMI, that may derive differential risks of CVD. However, there is limited evidence regarding the types of weight gain and cardiovascular outcomes in relation to weight gain in patients with COVID-19.

To address this evidence gap, we investigated the association of weight management with the risk of CVD after COVID-19 using a nationwide cohort.

## Methods

### Study design

Through the requirement of health insurance enrollment, the Korean National Health Insurance Service (NHIS) covers 97.2% of the country's population [13]. When the medical expenses are invoiced to it by medical institutions, every medical record—including health screening, qualifying, and treatment records—is gathered into the NHIS database. Only authorized researchers have access to the NHIS's gathered health-related data for any relevant study. Other descriptions of NHIS are provided elsewhere [14]. In order to actively advance the scientific basis for the prevention and treatment of infectious diseases, including COVID-19, the NHIS and the Korea Disease Control and Prevention Agency (KDCA) have established the K-COV-N cohort and matched COVID-19 patients to non-COVID-19 patients using propensity scores derived from age and sex and matched to a 1:10 ratio. Therefore, the 6.3 million people in the K-COV-N cohort data have the following characteristics: (1) demographic data (age, gender, income), medical data (diagnosis, treatment, drug prescriptions, interventions), health-related behavior (alcohol, smoking, exercise), and other clinical data from health screenings (serological data, anthropometric measurements) obtained between January 1, 2009, and December 31, 2021; (2) data on COVID-19 patients (age, sex, confirmed date, and transmission route).

### Study population

**Fig. 1:**
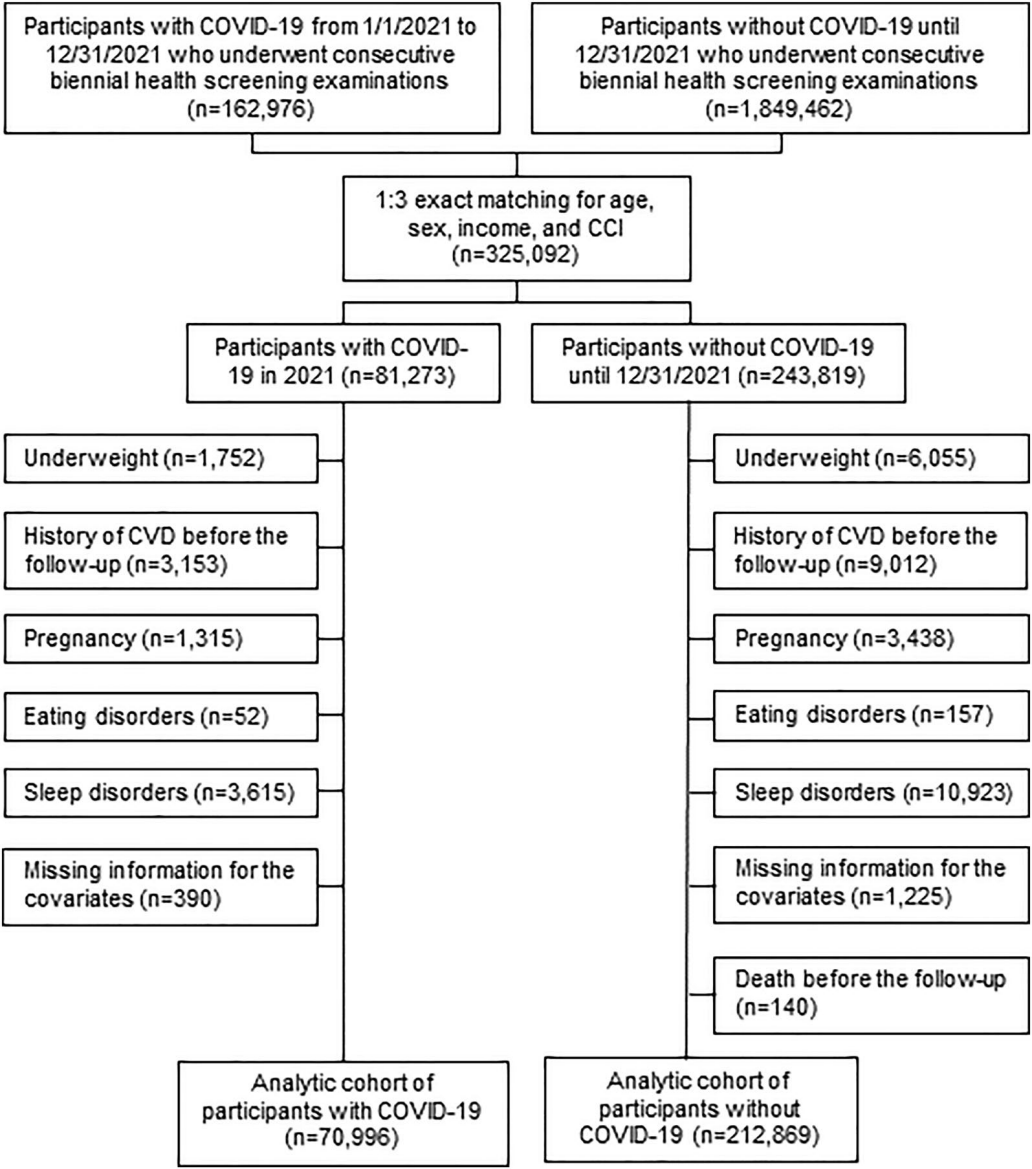
Flow diagram for the inclusion of study population

Participants in the South Korean health screening must be 20 years of age or older. Those who participated in both health screenings between period I (2017–2018) and period II (2019–2020) were initially included in the analysis (n = 2,024,728). Those who developed SARS-CoV-2 in 2021 were included in the COVID-19 group (n = 162,976), whereas those who did not receive a diagnosis of SARS-CoV-2 infection until December 31, 2021 were included in the control group (n = 1,849,462). The participants were exactly matched to a 1:3 ratio using age, sex, income, and Charlson Comorbidity Index (CCI) between the COVID-19 group and the control group. The income level was calculated by their monthly premium and was categorized as upper half or lower half. The International Classification of Diseases, 10th edition (ICD-10) codes used in the NHIS diagnosis data were used to determine CCI, which was then categorized as 0, 1, or 2. [15]. As a result, 81,273 participants of the COVID-19 group and 243,819 participants of the control group were matched. The index date was chosen for the COVID-19 group as the confirmed date of SARS-CoV-2 infection, and the matched control subjects used the same date. To avoid bias in the examination of the association, those with underweight (BMI < 18.5 kg/m^2^) from the COVID-19 (n = 1,752) and the control group (n = 6,055) were excluded [16]. Additionally, those with a history of CVD before the follow-up period were eliminated from the COVID-19 (n = 3,153) and control (n = 9,012) groups. Moreover, participants with pregnancy (n = 1,315), eating disorders (n = 52), sleep disorders (n = 3,615), other variables (n = 390) were not included in the COVID group. The participants from the control group excluded those with pregnancy (n = 3,438), eating disorders (n = 157), sleep disorders (n = 10,923), other variables (n = 1,225), or died before the follow-up (n = 140). Pregnancy, eating disorder, and sleep disorder was defined by the ICD-10 code of O, F50, F51, respectively. Finally, the analytic cohort contained 70,996 participants from the COVID-19 group and 212,869 participants from the control group (Fig. 1). This study was carried out in accordance with the STROBE recommendations and authorized by the Institutional Review Board of CHA University Hospital (No.: CHAMC 2022–05-052) [17]. The anonymity of the K-COV-N cohort data allowed for the waiver of the informed consent requirements.

### Exposures

BMI was measured by the weight (kg) divided by the height in meters squared from the biennial health screenings. Changes in BMI was defined by subtracting the BMI value from period II to I. If the change was within 1 kg/m^2^, we classified it as BMI stable. If the change was > 1 kg/m^2^ or > −1 kg/m^2^ then it was classified as BMI gain or BMI loss, respectively. In addition, to analyze the association of various factors that influence weight gain, we additionally calculated muscle mass index and fat mass index based on age, height, weight, waist circumference, and serum creatinine [9]. Therefore, the increase in muscle mass index and fat mass index was defined by the measurement from each period.

### Outcomes

The primary result of this study was an incident CVD after diagnosis of COVID-19. The incident CVD was defined based on the ICD-10 codes with at least 2 day of hospital admission or visit. The composite CVD events included any coronary heart disease (CHD; I20-I25) or stroke (I60-I69) occurrences [18]. Previous studies used same definition with our study using the NHIS database [19].

### Key variables

Age, sex, income, physical activity, alcohol consumption, smoking status, history of hypertension, diabetes mellitus, dyslipidemia, and CCI score from period II were used as covariates. Smoking status was categorized as current, former, and non-smoker. Alcohol consumption was categorized as a drinker or a non-drinker. Physical activity was calculated based on the frequency (day) of moderate- or vigorous-physical activity per week. The history of hypertension, diabetes, and dyslipidemia was defined by the record from medical experts during health screenings.

### Statistical analysis

To investigate the association of post COVID-19 CVD risk with weight change, we analyzed the association between BMI changes from period I (2017–2018) to period II (2019–2020) and the risk of CVD stratified by the diagnosis of COVID-19 and obesity. The participants with obesity were defined as those with BMI ≥ 25 kg/m^2^ following the Asia criteria of World Health Organization [16]. The eligible participants were followed-up from each index date until any event CVD diagnosis, death, or December 31, 2021, whichever happened first. For each continuous or categorical variable, the mean (standard deviation [SD]) or n (%) were provided, respectively. To compare the difference between groups, independent t-test analysis for continuous variables and chi-squared test for categorical data were used. In the primary analysis, we used Cox proportional hazard regression to determine the hazard ratio of BMI change with post COVID-19 CVD risk. The participants with stable BMI were used as reference value. Plotting the scaled Schoenfeld residuals were tested for a visual evaluation of the Cox regression model's proportionality assumption. In order to reduce the upwardly skewed risk estimates, we additionally computed subdistribution hazard ratio (sHR) using Fine-Gray subdistribution hazard model regression. The competing event was established as a pre-CVD death that occurred at least one month after SARS-CoV-2 infection, while the event of interest was set as an incident CVD. All covariates including age, sex, income, CCI, alcohol consumption, smoking status, history of diabetes mellitus, hypertension, and dyslipidemia were adjusted for the regression models. The regression models were stratified as follows: (1) COVID-19 patients without obesity, (2) COVID-19 patients with obesity, (3) non-COVID-19 patients without obesity, (4) non-COVID-19 patients with obesity. Both the sHR and the adjusted hazard ratio (aHR) were rounded to two decimal places. In addition, the participants that have factors that influence BMI gain (physical activity [active or inactive], comorbidity [no or moderate], increase in muscle mass index and fat mass index) were compared with those with stable BMI. To evaluate subgroup differences, we stratified the risk by age (65 or 65), history of hypertension (yes or no), diabetes (yes or no), and dyslipidemia (yes or no). All reported *P* values were two-sided and *P* values of < 0.05 were considered as statistically significant. All statistical analyses were performed using SAS 9.4 (SAS Institute, Cary, NC).

## Results

### Baseline characteristics

We identified 70,996 COVID-19 group and 212,869 control group in the analytic cohort. After the exclusion, there were 285 incident post COVID-19 CVD occurred from the analytic cohort. The mean BMI at period II was 24.8 (3.3) and 24.6 (3.3) for COVID-19 and no COVID-1 group, respectively. Similarly, the mean BMI at period I was 24.6 (3.3) and 24.4 (3.3) for COVID-19 and no COVID-1 group, respectively. The participants with COVID-19 exhibited higher physical activity levels, lower smoking rates, and a higher prevalence of hypertension, diabetes, and dyslipidemia history. However, these differences were not significant. The observed statistical significance seems attributable to the exceptionally large sample size. However, this minor difference implies little bias in the selection of the non-COVID-19 control group. Other baseline characteristics of two groups are described in Table 1.

**Table 1:** Baseline characteristics of the study participants

	No COVID-19 (n = 212,869)	COVID-19 (n = 70,996)	*P* value
At health screening period II (2019–2020)			
Age, years	56.4 (12.7)	56.5 (12.7)	0.491
Sex, n (%)			0.778
Men	107,267 (50.4)	35,819 (50.5)	
Women	105,602 (49.6)	35,177 (49.6)	
Household income, n (%)			0.722
Upper half	123,420 (58.0)	41,217 (58.1)	
Lower half	89,449 (42.0)	29,779 (41.9)	
BMI, kg/m^2^	24.6 (3.3)	24.8 (3.3)	< .001
ASMI, kg/m^2^	6.5 (0.6)	6.6 (0.6)	< .001
BFMI, kg/m^2^	8.4 (1.9)	8.5 (1.9)	< .001
Waist circumference, cm	83.1 (9.4)	83.7 (9.4)	< .001
Systolic blood pressure, mmHg	126.2 (14.6)	126.3 (14.6)	0.258
Diastolic blood pressure, mmHg	77.4 (9.9)	77.4 (10.0)	0.936
Total cholesterol, mg/dL	196.4 (41.2)	195.6 (41.0)	0.003
Triglyceride, mg/dL	132.8 (94.5)	129.4 (88.6)	< .001
Cigarette smoking, n (%)			< 0.001
Never	135,735 (63.8)	46,881 (66.0)	
Past	41,348 (19.4)	15,771 (22.2)	
Current	35,786 (16.8)	8,344 (11.8)	
Alcohol consumption, n (%)			< 0.001
Never	84,659 (39.8)	26,696 (37.6)	
Ever	128,210 (60.2)	44,300 (62.4)	
Physical activity, n (%)			< 0.001
None	57,081 (26.8)	18,464 (26.0)	
≤ 2 times/week	33,053 (15.5)	11,097 (15.6)	
3–4 times/week	41,372 (19.4)	13,706 (19.3)	
≥ 5 times/week	81,363 (38.2)	27,729 (39.1)	
Hypertension, n (%)	62,988 (29.6)	21,750 (30.6)	< 0.001
Diabetes, n (%)	25,706(12.1)	8,875 (12.5)	0.003
Dyslipidemia, n (%)	33,667 (15.8)	11,848 (16.7)	< 0.001
CCI, n (%)			0.848
0	82,032 (38.5)	27,274 (38.4)	
1	68,854 (32.4)	23,018 (32.4)	
≥ 2	61,983 (29.1)	20,704 (29.2)	
At health screening period I (2017–2018)			
BMI, kg/m^2^	24.4 (3.2)	24.6 (3.2)	< .001
ASMI, kg/m^2^	6.5 (0.6)	6.5 (0.6)	< .001
BFMI, kg/m^2^	8.3 (1.8)	8.4 (1.9)	< .001
Waist circumference, cm	82.4 (10.3)	82.9 (10.4)	< .001
Physical activity, n (%)			< .001
None	83,256 (39.1)	26,794 (37.7)	
≤ 2 times/week	37,421 (17.6)	12,404 (17.5)	
3–4 times/week	34,584 (16.3)	11,729 (16.5)	
≥ 5 times/week	57,608 (27.1)	20,069 (28.3)	
CCI, n (%)			0.767
0	110,257 (51.8)	36,852 (51.9)	
1	63,335 (29.8)	21,128 (29.8)	
≥ 2	39,277 (18.5)	13,016 (18.3)	
Cigarette smoking, n (%)			< .001
Never	132,548 (62.3)	45,878 (64.6)	
Past	29,963 (14.1)	11,635 (16.4)	
Current	50,358 (23.7)	13,483 (19.0)	

Data are presented as mean (standard deviation) for continuous and n (%) for categorical variables. Baseline characteristics were defined by the measurement at NHIS health screening period II (2019–20)

Acronyms: COVID-19, coronavirus disease 2019; BMI, body mass index; LBMI, lean body mass index; ASMI, appendicular skeletal muscle mass index; BFMI, body fat mass index; CCI, Charlson comorbidity index

The unexpectedly higher smoking rate of 16.8% in the non-COVID-19 group compared to 11.8% in the COVID-19 group contradicts expectations given smoking's substantial risk factor status for COVID-19 infection. Smokers may be less likely to seek medical attention or get tested for COVID-19, either because they have milder symptoms, or because they fear stigma or discrimination [20]. This could result in underreporting bias regarding the prevalence of smoking among COVID-19 patients. Therefore, the complete SARS-CoV-2 positivity rate among smokers in the entire dataset might have been underestimated owing to incomplete smoking status data. Moreover, bias in selection might influence the outcomes since smokers are prone to respiratory symptoms such as cough, expectoration, and sore throat, potentially prompting more frequent testing and a higher proportion of smokers showing negative SARS-CoV-2 results [21].

### Association of changes in BMI with risk of overall cardiovascular disease in the post COVID-19

**Fig. 2:**
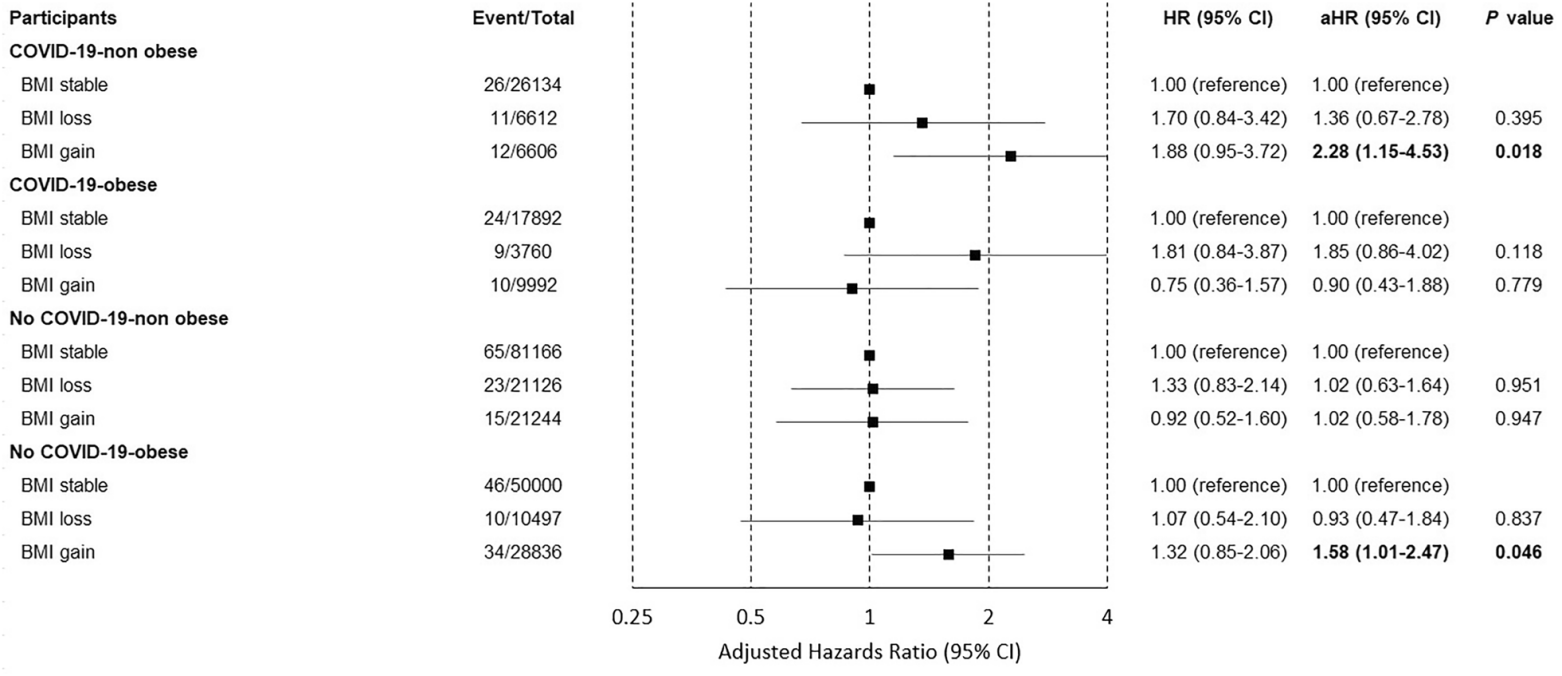
Forest plot of hazard ratios estimated by the BMI change with the risk of cardiovascular disease stratified by the diagnosis of COVID-19 and obesity. Adjusted hazard ratio was calculated using Cox proportional hazards regression after adjustments for age, household income, hypertension, diabetes mellitus, dyslipidemia, smoking, alcohol consumption, physical activity and the Charlson comorbidity index. COVID-19, coronavirus disease 2019; HR, hazard ratio; CI, confidence interval; aHR, adjusted hazard ratio

**Fig. 3:**
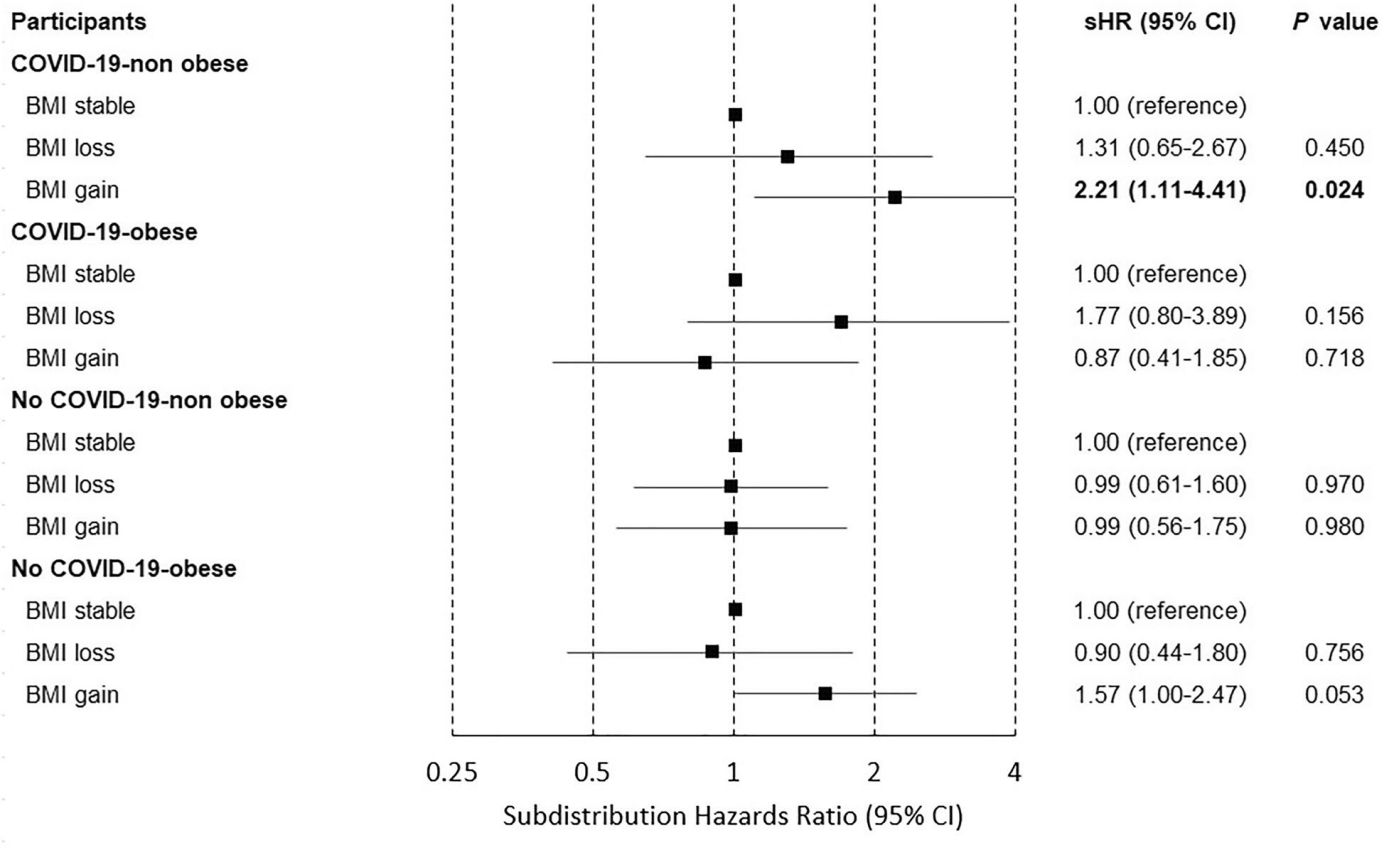
Forest plots of subdistribution hazard ratios estimated by the BMI change with the risk of cardiovascular disease stratified by the diagnosis of COVID-19 and obesity. Adjusted hazard ratio was calculated using Cox proportional hazards regression after adjustments for age, household income, hypertension, diabetes mellitus, dyslipidemia, smoking, alcohol consumption, physical activity and the Charlson comorbidity index. COVID-19, coronavirus disease 2019; HR, hazard ratio; CI, confidence interval; aHR, adjusted hazard ratio

Compared to those with stable BMI, those with BMI gain among COVID-19 patients without obesity (aHR, 2.28; 95% CI, 1.15–4.53;* P* value 0.018;) and non-COVID-19 patients with obesity (aHR, 1.58; 95% CI, 1.01–2.47; *P* value 0.046; Fig. 2) showed an increased risk of CVD. However, the results of the competing risk analysis only maintained the association with the participants among COVID-19 patients without obesity (sHR, 2.21; 95% CI, 1.11–4.41; *P* value 0.024; Fig. 3). This seems to be linked to a low occurrence rate of actual CVD events. To prevent an overestimation of the incidence rate of the event of interest in competing risk analysis, the cumulative incidence function is used, which requires an adequate number of observations for estimation [22]. If the incidence rate of a particular event is extremely low, the estimation of the cumulative incidence function for that event can become unstable, leading to wider confidence intervals [23, 24]. Consequently, it might be challenging to identify statistically significant differences. The association with coronary heart disease and stroke was not significant with BMI change (Additional file 1: Table S2).

### Association of changes in BMI with risk of overall cardiovascular disease in the post COVID-19 by various factors influencing BMI gain

Among those with COVID-19 patients without obesity, those who were physically inactive (aHR, 3.23; 95% CI, 1.26–8.29; *P* value 0.015), moderate comorbidities (aHR, 3.72; 95% CI, 1.68–8.26; *P* value 0.001), increased muscle mass index (aHR, 2.33; 95% CI, 1.17–4.63; *P* value 0.016), and increased fat mass index (aHR, 2.31; 95% CI, 1.16–4.59; *P* value 0.017) showed an increased risk for CVD. Similarly, among those with no-COVID-19-obese, those with moderate comorbidities (aHR, 1.89; 95% CI, 1.10–3.26; *P* value 0.022; Additional file 1: Table S3) and increased muscle mass index (aHR, 1.57; 95% CI, 1.00–2.45; *P* value 0.049; Table 2) showed an increased risk for CVD.

**Table 2:** Hazard ratios of factors influencing BMI gain with the cardiovascular disease risk stratified by the diagnosis of COVID-19 and obesity

Participants	Total	Event	PY	aHR (95% CI)	*P* value
COVID-19-non-obese					
BMI stable	26,134	26	2344	1.00 (reference)	
BMI gain					
With physical activity	4895	7	436	2.01 (0.88–4.61)	0.100
With physical inactivity	1711	5	152	3.23 (1.26–8.29)	0.015
With an increase in muscle mass index	6552	12	582	2.33 (1.17–4.63)	0.016
With an increase in fat mass index	6599	12	587	2.31 (1.16–4.59)	0.017
COVID-19-obese					
BMI stable	17,892	24	1593	1.00 (reference)	
BMI gain					
With physical activity	7258	5	647	0.70 (0.27–1.80)	0.457
With physical inactivity	2734	5	244	1.33 (0.51–3.46)	0.558
With an increase in muscle mass index	9915	10	882	0.89 (0.42–1.86)	0.750
With an increase in fat mass index	9981	10	889	0.88 (0.42–1.84)	0.726
No COVID-19-non-obese					
BMI stable	81,166	65	7325	1.00 (reference)	
BMI gain					
With physical activity	15,443	9	1358	0.96 (0.48–1.92)	0.913
With physical inactivity	5801	6	536	1.26 (0.56–2.86)	0.578
With an increase in muscle mass index	21,084	15	1879	1.05 (0.60–1.83)	0.877
With an increase in fat mass index	21,227	15	1892	1.03 (0.59–1.81)	0.909
No COVID-19-obese					
BMI stable	50,000	46	4493	1.00(reference)	
BMI gain					
With physical activity	20,687	23	1800	1.61 (0.97–2.67)	0.065
With physical inactivity	8149	11	723	1.53 (0.79–2.96)	0.203
With an increase in muscle mass index	28,644	34	2503	1.57 (1.00–2.45)	0.049
With an increase in fat mass index	28,807	34	2520	1.56 (0.99–2.43)	0.053

The participants were classified by the diagnosis of COVID-19 and obesity. The factors that influence weight gain include physical activity, comorbidity, muscle mass index, and fat mass index. For assessing the increase in muscle mass and fat mass index, the measurement from II (2019–2020) was subtracted from period I (2017–2018). aHR was calculated using Cox proportional hazards regression after adjustments for age, household income, hypertension, diabetes mellitus, dyslipidemia, smoking, alcohol consumption, physical activity, and the Charlson comorbidity index

Acronyms: COVID-19, coronavirus disease 2019; PY, person-years; HR, hazard ratio; CI, confidence interval; aHR, adjusted hazard ratio

### Subgroup analyses

Subgroup analyses were conducted by stratifying age, hypertension, diabetes, and dyslipidemia. The trend of increased CVD risk among COVID-19 patients without obesity was maintained with those with hypertension (aHR, 3.14; 95% CI, 1.31–7.52; *P* value 0.010; Additional file 1: Table S4). However, no significant differences were detected by other subgroups or other participant groups.

## Discussion

In individuals without obesity, who have COVID-19, the risk of developing CVD after COVID-19 in the group exposed to weight gain was significantly higher by 2.28 times compared to the weight maintenance group. On the other hand, in people with obesity without COVID-19, the risk of CVD incidence in the group exposed to weight gain was significantly higher by 1.58 times compared to the weight maintenance group. COVID-19 and obesity are both risk factors for CVD with established evidence. Even in people without obesity, weight gain can increase the risk of CVD, so it is important to manage your weight well. The results were consistent even in individuals with obesity, who do not have COVID-19. Moreover, the risk of CVD after COVID-19 appears to be higher among individuals without obesity who gain weight compared to those who maintain their weight. However, since obesity is a known risk factor for CVD incidence, the risk of developing CVD in the weight gain group of individuals with obesity compared to the weight maintenance group is relatively lower than that in individuals without obesity. Therefore, people who gained weight before COVID-19, whether obese or not, may be a high-risk group exposed to long-term CVD risk.

Our findings that COVID-19 patients who gained weight had a higher risk of CVD compared to those who maintained their weight, are in line with the well-established evidence linking weight gain to an increased risk of CVD. A study involving 73,435 non-hospitalized patients utilizing the health services of the US Department of Veterans Affairs revealed that all subgroups, including individuals with obesity, are at a higher risk of developing CVD after being infected with COVID-19 [3]. Furthermore, the group without obesity had a higher hazard ratio than the people with obesity when compared to the contemporary control group in terms of developing CVD after being infected with COVID-19 [25].

Chen et al. (2023) conducted a long-term cardiovascular study and found that COVID-19 did not increase the risk of CVD events, including heart failure, stroke, and myocardial infarction, despite adjusting for potential confounding factors such as age, gender, co-morbidities, and medications. However, the study did find a higher risk of CVD events among people who had obesity or diabetes before contracting COVID-19 [25]. Another study, which used data from the American Heart Association COVID-19 Cardiovascular Disease Registry, indicated that COVID-19 patients with obesity or had a higher BMI were more susceptible to cardiovascular events [26]. Nevertheless, the study did not address the combined impact of weight gain and CVD risk resulting from the complex interaction of factors such as various health behaviors, medical history, and weight categories associated with weight gain. In addition, previous studies mainly focused only on adults with overweight or obesity and did not consider weight change as a risk factor for CVD after COVID-19 in various populations, including populations without obesity [27].

The mechanism of weight gain and CVD risk is complicated and involves multiple factors [28], which is why people who are exposed to weight gain have a higher risk of developing CVD when infected with COVID-19 compared to those who maintain their weight. COVID-19 and CVD have a bidirectional relationship, but the exact cause of this interaction is not yet fully understood [29]. High levels of systemic inflammation due to COVID-19 can increase pre-existing CVD risk or cause new cardiovascular damage [18, 30]. COVID-19 is thought to cause inflammation and damage to the endothelial cells that line blood vessels, leading to blood clots that can cause heart attacks and strokes [31]. Studies conducted on animals suggest that ACE2, a surface protein that allows the virus to enter cells, and part of the renin–angiotensin–aldosterone system (RAAS), may be involved in this interaction [18, 30, 32].

Weight gain can increase the risk of developing CVD in general [33]. The mechanism of weight gain and CVD risk is complicated and involves multiple factors [6]. Our analysis of the NHIS cohort further extends the evidence for cardiovascular risk after COVID-19 associated with weight gain before COVID-19, using large population-level data and ascertained CVD events in the general population, including people with obesity.

A recent joint opinion from the World Heart Federation, American College of Cardiology, AHA, and the ESC emphasize that it is essential to provide unambiguous public health advice and strictly follow COVID-19 prevention procedures among all individuals with obesity [34]. The weight gain of individuals who are overweight or have a normal weight is not originally a major focus of research, unlike for individuals with obesity. However, our study stratified the population into two groups based on the risk factor of BMI(those with obesity and those without obesity) increase along with the CVD risk associated with COVID-19, and compared the risk of CVD due to weight gain with that of a group that maintained their weight. We suggest the importance of weight management for primary prevention of long-term CVD risk due to COVID-19 even in populations without obesity. The observational nature of our study prevents a definitive conclusion on the causal relationship between weight gain exposure, COVID-19, and CVD risk in the overall population, including obesity. Therefore, evidence for the effectiveness of weight management to alleviate CVD risk in population stratified by obesity status and COVID-19 needs to be further supported by controlled randomized studies.

## Limitations

We studied COVID-19’s long-term effects using real-world data, but had limitations. We couldn't gather individual weight change post-COVID-19 due to inconsistent body weight measurements, potentially causing errors and biases in estimating weight change and its link to cardiovascular outcomes. Utilizing pre-pandemic weight gain data from the last two years may not fully reflect the pandemic's impact on weight change and cardiovascular risk, potentially affecting our estimates of their association. Additionally, the follow-up time and the inability to stratify by COVID type can be considered limitations. In our study, we specifically evaluated the risk of cardiovascular disease during the short-term period following COVID-19, potentially explaining the observed low incidence. The emphasis on the follow-up time in our study aligns with previous research, consistently reporting a higher risk of cardiovascular disease within the first 30 days after COVID-19 infection [35]. However, the actual incidence and relative risk of CVD after COVID-19 infection are still uncertain, as data are limited and heterogeneous. Different data sources, methods, and definitions of COVID-19 and CVD may affect the estimates and comparisons of the long-term effects of COVID-19 on cardiovascular health [36].

Defining COVID type based on respiratory symptoms may not reflect infection severity or duration. Variability in viral variants or strains affecting clinical outcomes wasn't considered, limiting study population heterogeneity and generalizability to diverse settings or populations. The study did not account for the vaccination status of the COVID-19 patients, which may affect their prognosis and cardiovascular complications. Vaccination can lower the severity and mortality of COVID-19, and may also alter the obesity-cardiovascular disease link in COVID-19 patients [37]. At a similar time, the proportion of unvaccinated individuals among those infected with COVID-19 is approximately around 40% according to previous studies [38, 39]. Our NHIS claims data study extensively controlled for confounding factors (age, sex, habits, comorbidities, medications), boosting causal inference. Our population-based study covers 97% of Koreans, has high-quality validation and robustness. However, limitations persist in real-world data, including clinical coding ambiguity, incomplete symptom capture, and biases in claims data, demanding careful consideration for a comprehensive understanding of COVID-19's long-term effects.

## Conclusions

In conclusion, according to the NHIS cohort study, individuals with weight gain, including populations with overweight or obesity, as well as those with normal weight, had a significantly higher risk of developing the COVID-19-associated CVD than those who maintained their pre-pandemic weight. However, further studies are needed to evaluate the ongoing risk of COVID-19-related cardiovascular diseases resulting from weight gain in the general population. Long-term observational studies are needed for this purpose. Additionally, analyses that include other essential information such as vaccination status will be required.

### Supplementary Information

**Additional file 1.** Additional tables.

## Data Availability

No additional data available. Only authorized researchers received permission to access the National Health Insurance Service (NHIS) database at the Big Data Research Center of the Big Data Steering Department in the Republic of Korea.
