# Supplementary material for: COVID-19-related cardiovascular disease risk due to weight gain: a nationwide cohort study
Source: Eur J Med Res. 2024 Jan 2;29:2. doi: 10.1186/s40001-023-01569-7 (PMC10762936; doi:10.1186/s40001-023-01569-7)
Supplement: Supplementary file 1 — Additional file 1. Additional tables. [file 40001_2023_1569_MOESM1_ESM.docx]

**Table S1.** **Sensitivity analysis of hazard ratios estimated** **by** **the BMI change with the risk of cardiovascular disease stratified by the diagnosis of COVID-19 and obesity**

| **Participants** | **HR (95% CI)** | **aHR (95% CI)** | ***P* value** |
| --- | --- | --- | --- |
| COVID-19-non-obese |  |  |  |
| BMI stable | 1.00 (reference) | 1.00 (reference) |  |
| BMI loss | 0.76 (0.12-5.06) | 0.70 (0.10-4.72) | 0.711 |
| BMI gain | 0.80 (0.12-5.31) | 0.88 (0.13-5.95) | 0.893 |
| COVID-19-obese |  |  |  |
| BMI stable | 1.00 (reference) | 1.00 (reference) |  |
| BMI loss | 1.66 (0.46-6.00) | 1.49 (0.40-5.61) | 0.552 |
| BMI gain | 0.80 (0.25-2.57) | 0.99 (0.31-3.18) | 0.981 |
| No COVID-19-non-obese |  |  |  |
| BMI stable | 1.00 (reference) | 1.00 (reference) |  |
| BMI loss | 1.24 (0.61-2.54) | 1.03 (0.50-2.11) | 0.942 |
| BMI gain | 0.82 (0.35-1.94) | 0.91 (0.39-2.16) | 0.834 |
| No COVID-19-obese |  |  |  |
| BMI stable | 1.00 (reference) | 1.00 (reference) |  |
| BMI loss | 0.80 (0.25-2.56) | 0.75 (0.23-2.40) | 0.623 |
| BMI gain | 0.84 (0.38-1.85) | 0.93 (0.42-2.05) | 0.854 |

The participants were classified by the diagnosis of COVID-19 and obesity. Sensitivity analysis was performed by excluding the participants with less than one-month follow-up. aHR was calculated using Cox proportional hazards regression after adjustments for age, household income, hypertension, diabetes mellitus, dyslipidemia, smoking, alcohol consumption, physical activity, dose of COVID-19 vaccines, and the Charlson comorbidity index.

Acronyms: COVID-19, coronavirus disease 2019; HR, hazard ratio; CI, confidence interval; aHR, adjusted hazard ratio.

**Table S2.** **Hazard ratios estimated by** **the BMI change with the risk of coronary heart disease and stroke stratified by the diagnosis of COVID-19 and obesity**

|  | **Coronary heart disease** | | **Stroke** | |
| --- | --- | --- | --- | --- |
| **Participants** | **aHR (95% CI)** | ***P* value** | **aHR (95% CI)** | ***P* value** |
| COVID-19-non-obese |  |  |  |  |
| BMI stable | 1.00 (reference) |  | 1.00 (reference) |  |
| BMI loss | 1.45 (0.59-3.56) | 0.420 | 1.05 (0.32-3.46) | 0.938 |
| BMI gain | 2.14 (0.88-5.22) | 0.095 | 2.31 (0.78-6.83) | 0.129 |
| COVID-19-obese |  |  |  |  |
| BMI stable | 1.00 (reference) |  | 1.00 (reference) |  |
| BMI loss | 1.08 (0.36-3.24) | 0.895 | 4.40 (1.30-14.85) | 0.017 |
| BMI gain | 0.67 (0.26-1.71) | 0.670 | 1.73 (0.52-5.70) | 0.370 |
| No COVID-19-non-obese |  |  |  |  |
| BMI stable | 1.00 (reference) |  | 1.00 (reference) |  |
| BMI loss | 1.17 (0.61-2.25) | 0.637 | 0.88 (0.43-1.79) | 0.717 |
| BMI gain | 0.65 (0.25-1.67) | 0.369 | 1.30 (0.64-2.63) | 0.473 |
| No COVID-19-obese |  |  |  |  |
| BMI stable | 1.00 (reference) |  | 1.00 (reference) |  |
| BMI loss | 1.27 (0.54-2.97) | 0.587 | 0.52 (0.16-1.76) | 0.296 |
| BMI gain | 1.60 (0.86-3.00) | 0.140 | 1.54 (0.81-2.92) | 0.186 |

The participants were classified by the diagnosis of COVID-19 and obesity. aHR was calculated using Cox proportional hazards regression after adjustments for age, household income, hypertension, diabetes mellitus, dyslipidemia, smoking, alcohol consumption, physical activity, and the Charlson comorbidity index.

Acronyms: COVID-19, coronavirus disease 2019; HR, hazard ratio; CI, confidence interval; aHR, adjusted hazard ratio.

**Table S3. Hazard ratios of factors influencing BMI gain with the cardiovascular disease risk stratified by** **the diagnosis of COVID-19 and obesity**

| **Participants** | **Total** | **Event** | **PY** | **aHR (95% CI)** | ***P* value** |
| --- | --- | --- | --- | --- | --- |
| COVID-19-non-obese |  |  |  |  |  |
| BMI stable | 26134 | 26 | 2344 | 1.00 (reference) |  |
| BMI gain |  |  |  |  |  |
| With moderate comorbidities | 1537 | 8 | 146 | 3.72 (1.68-8.26) | 0.001 |
| Without comorbidities | 2968 | 3 | 256 | 2.23 (0.70-7.15) | 0.177 |
| COVID-19-obese |  |  |  |  |  |
| BMI stable | 17892 | 24 | 1593 | 1.00 (reference) |  |
| BMI gain |  |  |  |  |  |
| With moderate comorbidities | 2803 | 7 | 263 | 1.43 (0.61-3.33) | 0.413 |
| Without comorbidities | 3878 | 2 | 334 | 0.81 (0.21-3.15) | 0.813 |
| No COVID-19-non-obese |  |  |  |  |  |
| BMI stable | 81166 | 65 | 7325 | 1.00 (reference) |  |
| BMI gain |  |  |  |  |  |
| With moderate comorbidities | 5002 | 9 | 488 | 1.43 (0.71-2.86) | 0.318 |
| Without comorbidities | 9281 | 2 | 793 | 0.67 (0.18-2.43) | 0.540 |
| No COVID-19-obese |  |  |  |  |  |
| BMI stable | 50000 | 46 | 4493 | 1.00 (reference) |  |
| BMI gain, |  |  |  |  |  |
| With moderate comorbidities | 7903 | 19 | 746 | 1.89 (1.10-3.26) | 0.022 |
| Without comorbidities | 11484 | 3 | 956 | 0.64 (0.21-1.99) | 0.444 |

The participants were classified by the diagnosis of COVID-19 and obesity. The factors that influence weight gain include physical activity, comorbidity, muscle mass index, and fat mass index. For assessing the increase in muscle mass and fat mass index, the measurement from II (2019-2020) was subtracted from period I (2017-2018). aHR was calculated using Cox proportional hazards regression after adjustments for age, household income, hypertension, diabetes mellitus, dyslipidemia, smoking, alcohol consumption, physical activity, and the Charlson comorbidity index.

Acronyms: COVID-19, coronavirus disease 2019; PY, person-years; HR, hazard ratio; CI, confidence interval; aHR, adjusted hazard ratio.

**Table S4.** **Subgroup analysis for the hazard ratios estimated by** **the BMI gain with the risk of cardiovascular disease stratified by the diagnosis of COVID-19 and obesity**

| **Subgroups** | **BMI stable** | **COVID-19-non-obese** | | | **COVID-19-obese** | | | **No COVID-19-non-obese** | | | **No COVID-19-obese** | | |
| --- | --- | --- | --- | --- | --- | --- | --- | --- | --- | --- | --- | --- | --- |
|  |  | **BMI gain** | ***P* value** | ***P-*interaction** | **BMI gain** | ***P* value** | ***P-*interaction** | **BMI gain** | ***P* value** | ***P-*interaction** | **BMI gain** | ***P* value** | ***P-*interaction** |
| Age |  |  |  | 0.094 |  |  | 0.675 |  |  | 0.652 |  |  | 0.652 |
| ≥65 | 1.00 (reference) | 3.45 (1.54-7.70) | 0.003 |  | 1.04 (0.40-2.70) | 0.931 |  | 1.14 (0.57-2.23) | 0.743 |  | 1.14 (0.57-2.23) | 0.743 |  |
| <65 | 1.00 (reference) | 0.88 (0.21-3.67) | 0.858 |  | 0.74 (0.23-2.36) | 0.612 |  | 0.90 (0.35-2.31) | 0.818 |  | 0.90 (0.35-2.31) | 0.818 |  |
| Hypertension |  |  |  | 0.350 |  | 0.916 | 0.686 |  |  | 0.422 |  |  | 0.422 |
| Yes | 1.00 (reference) | 3.14 (1.31-7.52) | 0.010 |  | 1.05 (0.43-2.57) |  |  | 0.82 (0.35-1.92) | 0.649 |  | 0.82 (0.35-1.92) | 0.649 |  |
| No | 1.00 (reference) | 1.57 (0.52-4.77) | 0.627 |  | 0.75 (0.21-2.72) |  |  | 1.32 (0.63-2.76) | 0.461 |  | 1.32 (0.63-2.76) | 0.461 |  |
| Diabetes |  |  |  | 0.393 |  |  | 0.260 |  |  | 0.152 |  |  | 0.152 |
| Yes | 1.00 (reference) | 3.68 (0.86-15.78) | 0.080 |  | 2.13 (0.46-9.90) | 0.334 |  | 1.91 (0.68-5.35) | 0.218 |  | 1.91 (0.68-5.35) | 0.218 |  |
| No | 1.00 (reference) | 2.05 (0.94-4.46) | 0.071 |  | 0.72 (0.31-1.70) | 0.458 |  | 0.84 (0.43-1.64) | 0.614 |  | 0.84 (0.43-1.64) | 0.614 |  |
| Dyslipidemia |  |  |  | 0.057 |  |  | 0.737 |  |  | 0.727 |  |  | 0.727 |
| Yes | 1.00 (reference) | 9.42 (1.72-51.69) | 0.010 |  | 1.03 (0.18-5.78) | 0.972 |  | 0.88 (0.27-2.88) | 0.841 |  | 0.88 (0.27-2.88) | 0.841 |  |
| No | 1.00 (reference) | 1.30 (0.78-2.19) | 0.315 |  | 1.65 (0.75-3.67) | 0.216 |  | 0.87 (0.38-1.96) | 0.731 |  | 0.87 (0.38-1.96) | 0.731 |  |

The participants were classified by the diagnosis of COVID-19 and obesity. aHR was calculated using Cox proportional hazards regression after adjustments for age, household income, hypertension, diabetes mellitus, dyslipidemia, smoking, alcohol consumption, physical activity, and the Charlson comorbidity index.

Acronyms: COVID-19, coronavirus disease 2019; HR, hazard ratio; CI, confidence interval; aHR, adjusted hazard ratio.
